# Effect of AlN Cap Layer on Polarization Coulomb Field Scattering in AlGaN/GaN Heterostructure Field Effect Transistor

**DOI:** 10.3390/mi16101093

**Published:** 2025-09-26

**Authors:** Qianding Cheng, Ming Yang, Zhiliang Gao, Ruojue Wang, Jihao He, Feng Yan, Xu Tang, Weihong Zhang, Zijun Hu, Jingguo Mu

**Affiliations:** 1 Beijing Orient Institute of Measurement and Test, Beijing 100094, China; 2 Beijing Engineering Laboratory of Electrostatic Protection and Application Technology for Electronic Products, Beijing 100094, China

**Keywords:** AlGaN/GaN HFETs, AlN cap layer, polarization Coulomb field scattering, electron mobility

## Abstract

In this study, AlGaN/GaN heterostructure field-effect transistors (HFETs) with an AlN cap layer and a GaN cap layer were fabricated. The devices were of different sizes. Capacitance–voltage (*C-V*) and current–voltage (*I-V*) curves were measured. Based on two-dimensional (2D) scattering theory, electron mobility corresponding to polarization Coulomb field (PCF) scattering and other primary scattering mechanisms was quantitatively determined. The influence of the AlN cap layer on PCF scattering in AlGaN/GaN HFETs was studied. It was found that the AlN cap layer suppresses the inverse piezoelectric effect (IPE) in the AlGaN barrier layer because of its greater polarization and larger Young’s modulus, thereby reducing the generation of additional polarization charge (APC) under the gate. In addition, the 2D electron gas (2DEG) density (*n*_2DEG_) under the gate of the samples with an AlN cap layer is higher. Both factors help reduce PCF scattering intensity. Moreover, mobility analysis of samples with different gate–drain spacings (*L*_GD_) showed that PCF scattering is less affected by *L*_GD_ variations in devices with AlN cap layers. This study offers new insights into the structural optimization of AlGaN/GaN HFETs.

## 1. Introduction

GaN is widely recognized as a key material for high-power electronic applications owing to its wide bandgap and high electron saturation velocity [1,2,3]. Based on GaN, AlGaN/GaN HFETs exhibit excellent voltage resistance and high-frequency characteristics, showing significant potential in mobile communication and power electronics [4,5]. In AlGaN/GaN HFETs, PCF scattering has been identified as a major scattering mechanism in previous studies [6,7,8]. It is induced by the polarization effect generated by AlGaN/GaN HFETs, an effect that significantly influences 2DEG mobility [6,7]. Regulating the polarization effect remains a key challenge in achieving breakthrough AlGaN/GaN HFET performance [5,9].

A cap layer is critical for device performance and reliability, including in terms of polarization effect regulation, threshold voltage modulation, and improvement of ohmic contacts. A GaN cap layer is frequently utilized as a standard configuration in AlGaN/GaN HFET structures. Since both the spontaneous and piezoelectric polarization strength of AlN are significantly higher than those of GaN [10], an AlN cap layer can increase the channel *n*_2DEG_ and alter electric field strength beneath the gate. These factors can improve device performance and influence PCF scattering intensity. Although researchers have conducted extensive research on PCF scattering, these studies have mainly focused on GaN HFETs with conventional GaN cap layers and only elucidated the effect of the GaN cap layer on PCF scattering [6,7,8,11]. Clarifying the effect of the AlN cap layer on PCF scattering is crucial for future device structure design and performance optimization. However, the influence of an AlN cap layer on PCF scattering is unclear, so further investigation is necessary in this regard. Such research is critical for optimizing the structures of materials and devices with respect to the AlN cap layer, thereby improving performance metrics such as the saturation current.

In this research, AlGaN/GaN HFETs of different sizes with AlN and GaN cap layers were fabricated. Based on the measured electrical data, the 2DEG mobility was calculated, and the influence of the AlN cap layer on PCF scattering in AlGaN/GaN HFETs was systematically analyzed.

## 2. Experimental Section

**Figure 1:**
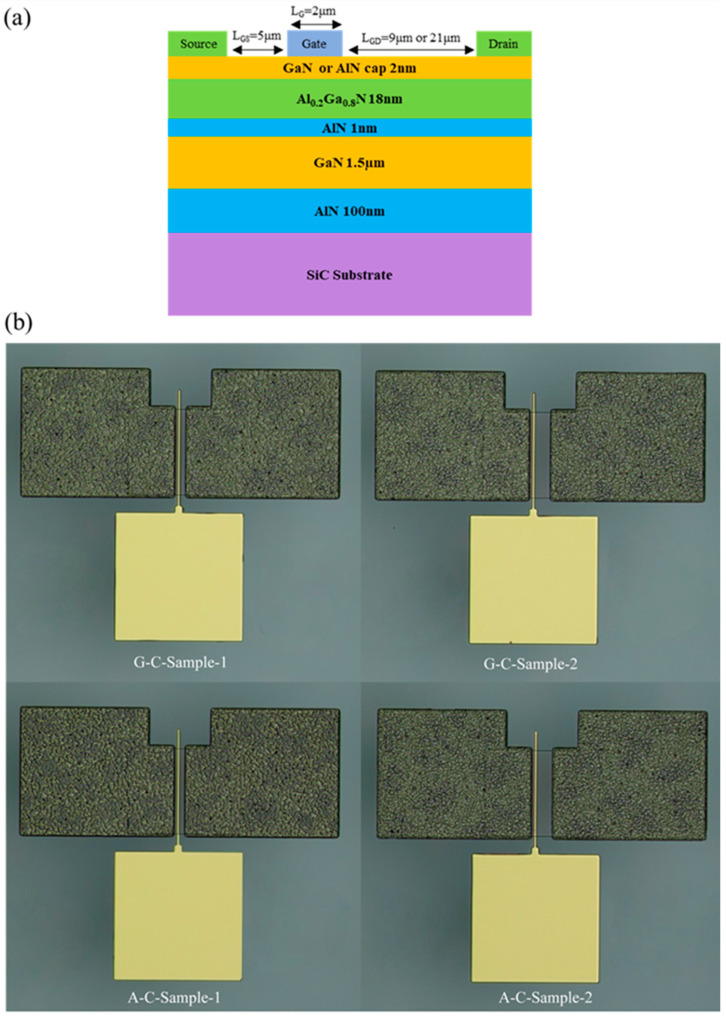
(**a**) Schematic diagram of the material and device structure (**b**) Microscopic images of four samples.

As shown in Figure 1a, the material composition of the AlGaN/GaN wafer used to manufacture the device is as follows: a 4H-SiC substrate, a 100 nm AlN layer, a 1500 nm GaN layer, a 1 nm AlN intercalation layer, a 18 nm Al_0.2_Ga_0.8_N layer, and a 2 nm GaN/AlN layer. The fabrication of the AlGaN/GaN HFET began with defining the device mesa using inductively coupled plasma (ICP) etching. The ICP etching time was 15 min, and the mesa-etching depth was 150 nm. For the source and drain electrodes, Ti/Al/Ni/Au multilayer metals with thicknesses of 30 nm, 150 nm, 50 nm, and 50 nm were deposited via magnetron sputtering. Rapid thermal annealing was then carried out at 850 °C for 40 s under a high-purity nitrogen ambient atmosphere to form alloyed ohmic contacts. The Schottky gate was formed by depositing a Ni/Au bilayer metal, with each layer having a thickness of 100 nm, to ensure clear rectifying characteristics. The fabricated devices featured a gate length (*L*_G_) of 2 μm and a gate-source distance (*L*_GS_) of 5 μm. The *L*_GD_ was designed with two configurations: 9 μm (*L*_GD1_) and 21 μm (*L*_GD2_). The GaN cap layer devices with 9 μm *L*_GD_ and 21 μm *L*_GD_ were dubbed G-C-Sample-1 and G-C-Sample-2. The AlN cap layer devices with 9 μm *L*_GD_ and 21 μm *L*_GD_ were dubbed A-C-Sample-1 and A-C-Sample-2. The gate width (*W*) for all samples was 100 μm. Microscopic images of the four samples are shown in Figure 1b. The device’s *C-V* and *I-V* responses were measured.

## 3. Results and Discussion

**Figure 2:**
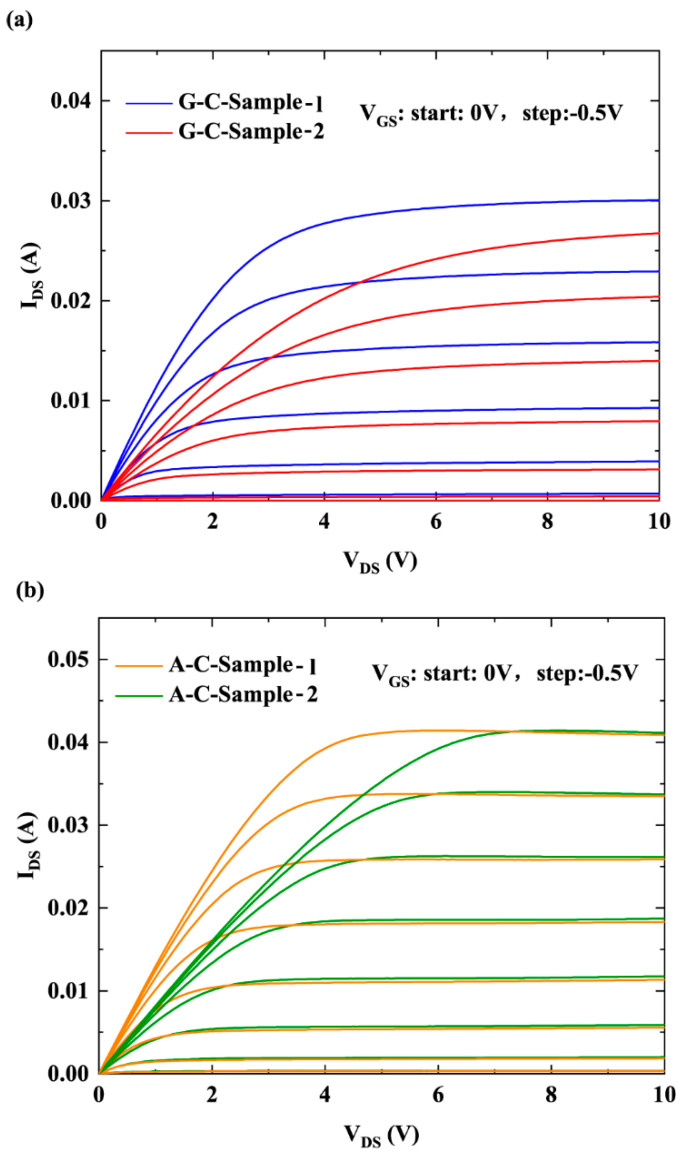
*I-V* measurements of samples with (**a**) GaN and (**b**) AlN cap layers.

Figure 2 presents the *I-V* measurement curves for four samples. The channel current (*I*_DS_) corresponding to gate voltage (*V*_GS_) when the gate–drain bias was 0.1 V was selected to calculate the 2DEG mobility. To fully account for the variation in the 2DEG density under the gate induced by *V*_GS_ and improve calculation accuracy, the 2DEG channel was divided into three electronic systems: the under-gate electron system, the gate–source electron system, and the gate-drain electron system. The 2DEG mobility of the under-gate electron system was determined through iterative calculation. The detailed procedure for calculating the 2DEG mobility corresponds to Method 1 described in [12,13]. The 2DEG mobility corresponding to each scattering mechanism is obtained by first calculating the momentum relaxation time. For PCF scattering, the scattering process of 2DEG electrons is represented by scattering matrix elements. The scattering matrix element from the initial state ***k*** to the final state ***k*’** is written as [12,13]:

(1) $$\begin{array}{l} M_{k\to k^{\prime }}\\ =A^{-1}\int_{0}^{\infty }\psi_{k\prime }^{*}(z)[\int_{a}^{b}dx\int_{0}^{W}V\left(x,y,z\right)\exp\left(-iq_{x}x-iq_{y}y\right)dy]\psi_{k}(z)dz\\ =A^{-1}\int_{0}^{\infty }\psi_{k\prime }^{*}(z)[V(q_{x},q_{y},z)]\psi_{k}(z)dz. \end{array}$$

*q*_x_ and *q*_y_ are the components of the wave vector ***q*** in the x and the y direction. The integral interval (a, b) of *x* is the coordinate interval of the channel region in which the 2DEG electrons are located in the *x* direction. The integration intervals are (−*L*_G_/2, *L*_G_/2) when calculating the 2DEG mobility of under-gate electron system. ***q*** = ***k*** − ***k’*** indicates the change in the wave vector in the scattering process.

The energy-dependent rate of PCF scattering is [12,13]:

(2) $$\frac{1}{\tau_{\mathrm{PCF}}\left(E\right)}=\frac{Am^{*}}{2\pi \hbar^{3}}\int_{-\pi }^{\pi }{\left|\frac{M_{k\to k^{\prime }}}{S\left(q,T_{e}\right)}\right|}^{2}\left(1-\cos\theta \right)\;d\theta .$$

θ is the scattering angle from ***k*** to ***k*’**, *T*_e_ is the 2DEG electron temperature. *S* (*q*, *T*_e_) is written as [12,13]:

(3) $$S(q,T_{e})=1+\frac{e^{2}F(q)\Pi (q,T_{e},E)}{2\varepsilon_{0}\varepsilon_{s}q}.$$

*F* (*q*) is the form factor [12,13]:

(4) $$F(q)=\int_{0}^{\infty }\int_{0}^{\infty }\psi^{2}\left(z\right)\psi^{2}(z^{\prime })\exp(-q\left|z-z^{\prime }\right|)dzdz^{\prime }$$

The polarizability function Π (*q*, *T*_e_, *E*) is written as [12,13]:

(5) $$\Pi (q,T_{e},E)=\frac{m^{*}}{4\pi \hbar^{2}k_{B}T_{e}}\int_{0}^{\infty }\frac{1-\Theta (q-2k_{F}){\left[1-{\left(2k_{F}/q\right)}^{2}\right]}^{1/2}}{\cosh^{2}[\left(E_{F}-E\right)/2k_{B}T_{e}]}dE$$

where $\;\Theta \left(x\right)$ is the usual step function, $k_{F}=\;{\left(2\pi n_{2D}\right)}^{1/2}$ is the Fermi wave vector, and *E*_F_ is the Fermi energy.

Finally, based on the Fermi distribution, the momentum relaxation time of PCF scattering is obtained as [12,13]:

(6) $$\tau_{\mathrm{PCF}}=\int \tau_{\mathrm{PCF}}(E)E\frac{\partial f_{0}(E)}{\partial E}dE/\int E\frac{\partial f_{0}(E)}{\partial E}dE$$

where *f*_0_ is the Fermi distribution function, which can be written as [12,13]:

(7) $$f_{0}\left(E\right)=\frac{1}{\exp\left[\left(E-E_{F}\right)/k_{B}T_{e}\right]+1}$$

where *k*_B_ is the Boltzmann constant.

For polar optical phonon (POP) scattering, the momentum relaxation time is [13]:

(8) $$\tau_{POP}=\frac{2\varepsilon^{*}k_{0}\hbar^{2}P_{POP}(y)}{e^{2}\omega_{POP}m^{*}N_{B}(T)G(k_{0})}$$

where $\varepsilon^{*}=\varepsilon_{0}/(1/\varepsilon_{h}-1/\varepsilon_{s})$, $k_{0}=\sqrt{2m^{*}(\hbar \omega_{POP})/\hbar^{2}}$, $\hbar \omega_{POP}$ is the polar optical phonon energy $P_{POP}(y)=1+(1-e^{-y})/y$, $y=\pi \hbar^{2}n_{2DEG}/(m^{*}k_{B}T)$, $N_{B}(T)=1/[\exp(\hbar \omega_{POP}/k_{B}T)-1]$ is the Bose–Einstein distribution function.

For piezoelectric (PE) scattering, the momentum relaxation time is [13]:

(9) $$\tau_{PE}={\left[\frac{e^{2}M^{2}k_{B}Tm^{*}}{4\pi \varepsilon_{0}\varepsilon_{s}\hbar^{3}k_{F}^{3}}\int_{0}^{2k_{F}}\frac{F(k)k^{3}}{{\left[k+k_{\mathrm{TF}}F(k)\right]}^{2}\sqrt{1-{\left[k/\left(2k_{F}\right)\right]}^{2}}}\;dk\right]}^{-1}$$

where *M*^2^ is the electromechanical coupling coefficient, $q_{\mathrm{TF}}=m^{*}e^{2}/\left(2\pi \varepsilon_{0}\varepsilon_{s}\hbar^{2}\right)$ is the Thomas-Fermi wave vector, the change in wave vector before and after scattering:

(10) $$k=2k_{F}\sin(\theta /2),k_{F}=\sqrt{2\pi n_{2DEG}},\;\theta \in (0,\pi )$$

For acoustic deformation potential (DP) scattering, the momentum relaxation time is [13]:

(11) $$\tau_{DP}=\frac{16\rho \nu_{s}^{2}\hbar^{3}}{3m^{*}a_{c}^{2}k_{B}Tb}$$

where $a_{c}$ is acoustic deformation potential, $b={\left(33m^{*}e^{2}n_{2\mathrm{DEG}}/8\varepsilon_{s}\hbar^{2}\right)}^{1/3}$, $\nu_{s}$ is the longitudinal acoustic wave velocity of GaN.

For dislocation (DIS) scattering, the momentum relaxation time is [13]:

(12) $$\tau_{DIS}={\left[\frac{N_{\mathrm{DIS}}m^{*}e^{2}\rho_{L}^{2}}{\hbar^{3}\varepsilon_{0}^{2}\varepsilon_{s}^{2}}\left(\frac{1}{16\pi k_{F}^{4}}\right)\int_{0}^{1}\frac{du}{{\left(u+\frac{q_{\mathrm{TF}}}{2k_{F}}\right)}^{2}\sqrt{1-u^{2}}}\right]}^{-1}$$

where $N_{\mathrm{DIS}}$ is the dislocation density, $\rho_{L}=ef_{DIS}/c_{0}$, $f_{\mathrm{DIS}}$ is the probability that the dislocation energy state is filled, *c*_0_ is the lattice constant of GaN in the (0001) direction GaN.

For interface roughness (IFR) scattering, the momentum relaxation time is [13]:

(13) $$\tau_{IFR}={\left[\frac{\Delta^{2}L^{2}e^{4}m^{*}}{2{\varepsilon_{0}}^{2}{\varepsilon_{s}}^{2}\hbar^{3}}{\left(\frac{1}{2}n_{2DEG}\right)}^{2}\int_{0}^{1}\frac{u^{4}\exp\left(-k_{F}^{2}L^{2}u^{2}\right)}{{\left[u+G(k)q_{\mathrm{TF}}/\left(2k_{F}\right)\right]}^{2}\sqrt{1-u^{2}}}du\right]}^{-1}$$

where Δ is the root mean square roughness; *L* is the correlation length, $u=k/(2k_{F})$, $k_{F}$ is the Fermi wave vector.

According to the above equation, the momentum relaxation time of PCF scattering and other major scattering can be obtained. Under the momentum relaxation approximation, the relationship between the electron mobility and momentum relaxation time corresponding to each scattering is [12,13]:

(14) $$\mu =e\tau /m^{*}$$

Therefore, the 2DEG mobility corresponding to PCF scattering and other main scattering is obtained. According to Matheissen’s rule, the total 2DEG mobility and the mobility corresponding to other main scattering satisfy the relationship [12,13]:

(15) $$\frac{1}{\mu_{Total}}=\frac{1}{\mu_{PCF}}+\frac{1}{\mu_{POP}}+\frac{1}{\mu_{PE}}+\frac{1}{\mu_{DP}}+\frac{1}{\mu_{DIS}}+\frac{1}{\mu_{IFR}}$$

Therefore, the 2DEG mobility corresponding to each scattering mechanism and total electron mobility are calculated based on the above 2D scattering theory. Then, the total resistance *R* of the 2DEG channel is calculated based on the relationship between 2DEG mobility and resistance. On the other hand, based on the measured channel current when *V*_DS_ is 0.1 V, the total resistance *R*_Total_ of the 2DEG channel is calculated using Ohm’s law and ohmic contact resistance. When *R* = *R*_Total_, the iteration is stopped and the value of the 2DEG mobility is obtained.

**Figure 3:**
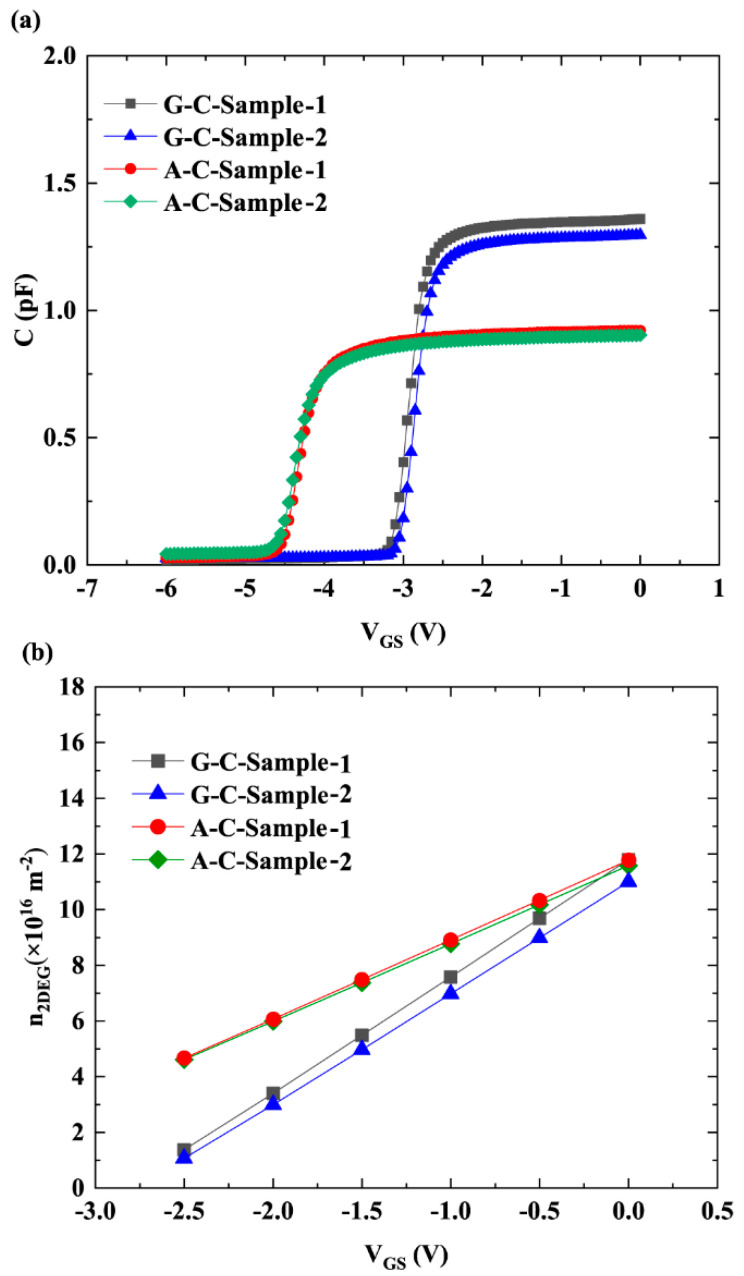
(**a**) Experimentally obtained *C-V* data and (**b**) calculated values of 2DEG density.

Figure 3a illustrates the *C-V* of the gate Schottky contact. G-C-Sample-1/2 and A-C-Sample-1/2 were fabricated using the same materials and identical gate areas. With good process uniformity, only slight differences were observed between the two samples within each group. In contrast, the differences between the G-C and A-C samples originate from the different cap layer materials. Based on *C-V* data, the *n*_2DEG_ under the gate for each sample, as shown in Figure 3b, was obtained using the following formula [14,15,16,17,18]:

(16) $$n_{2\mathrm{DEG}}=\int_{V_{\mathrm{TH}}}^{V_{\mathrm{GS}}}\frac{CdV}{L_{G}\cdot W\cdot e}$$

where *C* is the capacitance, as shown in Figure 3a; *e* is electron charge; and V_TH_ is threshold voltage. It is evident from Figure 3b that, for the two samples with the same cap layer, the 2DEG densities are similar because the gate area is the same. The samples with an AlN cap layer exhibit higher 2DEG density than those with a GaN cap layer. This is because the stronger spontaneous polarization of AlN introduces a higher polarization charge density in AlGaN while also introducing tensile strain that further enhances the piezoelectric polarization of AlGaN. This tensile strain, in combination with high spontaneous polarization of AlN, significantly increases the total polarization charge, thereby increasing 2DEG density [19,20]. Since PCF scattering is Coulomb scattering, it is subject to Coulomb screening. The screening factor is expressed as Equation (3). A higher *n*_2DEG_ indicates a more pronounced Coulomb-screening effect, which weakens the intensity of PCF scattering. Therefore, the sample with the AlN cap layer, which has a higher *n*_2DEG_, exhibited weaker PCF scattering.

**Figure 4:**
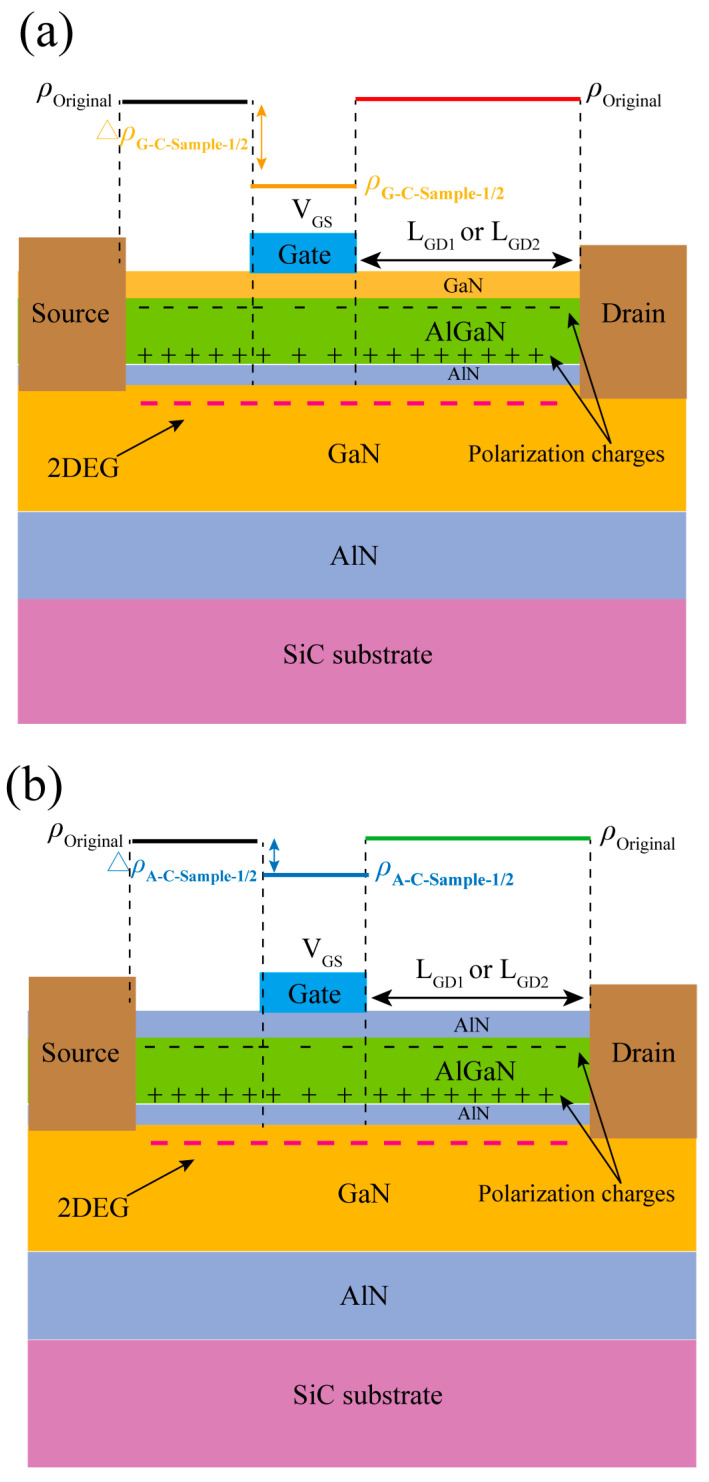
Illustration of the polarization charge distribution for (**a**) G-C-Sample-1/2, (**b**) A-C-Sample-1/2.

Moreover, compared with GaN cap layer, the AlN cap layer significantly increases the energy band at the top of the barrier layer, forming a higher electron barrier and reducing the gate voltage’s ability to regulate the electric field within the barrier layer. The strong polarization effect of the AlN cap layer prevents the external electric field from penetrating the AlGaN barrier layer, reducing the vertical electric field strength within the barrier layer. In addition, AlN has a higher Young’s modulus (AlN: 339 GPa, GaN: 261 GPa) [21], which makes it less susceptible to deformation and limits the strain amplitude of the barrier layer, thereby reducing the intensity of the IPE of AlGaN. These factors reduce the IPE of AlGaN, resulting in less APC under the gate when applying the same *V*_GS_. Figure 4 illustrates the polarization charge distribution for the four samples. In this figure, *ρ*_Original_ represents the original polarization charge density of the material when no gate bias is applied. *ρ*_G-C-Sample-1/2_ (*ρ*_A-C-Sample-1/2_) is the polarization charge with *V*_GS_, and ∆*ρ*_G-C-Sample-1/2_ (∆*ρ*_A-C-Sample-1/2_) represents the change in polarization charge due to *V*_GS_. Figure 4a and Figure 4b demonstrate the differences in APC induced by the GaN and AlN cap layers, respectively.

**Figure 5:**
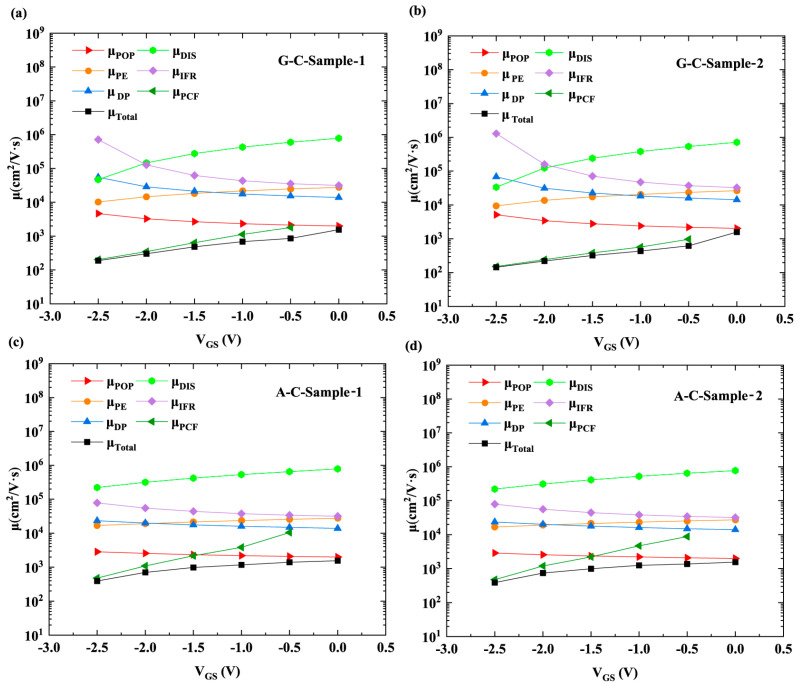
Electron mobility for (**a**) G-C-Sample-1, (**b**) G-C-Sample-2, (**c**) A-C-Sample-1, and (**d**) A-C-Sample-2.

**Figure 6:**
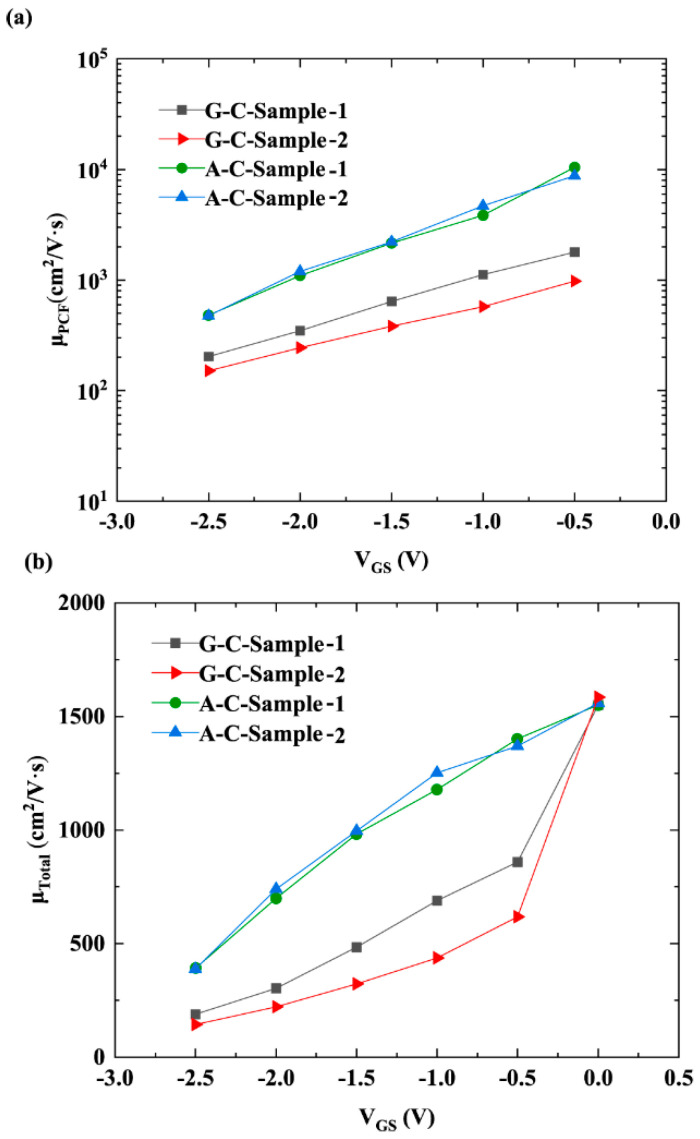
(**a**) μ_PCF_ and (**b**) μ_Total_ as a function of *V*_GS_ for the four samples.

As mentioned above, when AlN is used as the cap layer of AlGaN/GaN HFETs, the increase in *n*_2DEG_ and reduction in APC under the gate both contribute to a reduction in PCF-scattering intensity. Figure 5 shows the 2DEG mobility corresponding to PCF scattering (μ_PCF_), POP scattering (μ_POP_), PE scattering (μ_PE_), DP scattering (μ_DP_), DIS scattering (μ_DIS_), IFR scattering (μ_IFR_), and total electron mobility (μ_Total_). Detailed information on the calculation methods and corresponding equations for the 2DEG mobility associated with these scattering mechanisms can be found in [12,13]. Figure 5 reveals that PCF scattering exhibits lower electron mobility, which has an important influence on the total mobility and variation trend of *V*_GS_ according to the Matthiessen rule [22]. Figure 6a,b show μ_PCF_ and μ_Total_. Since the IPE does not occur at a gate bias of 0 V, no APC or PCF scattering is generated. Therefore, the μ_PCF_ at a gate bias of 0 V is not plotted in Figure 6a. As illustrated in Figure 6a, for the two GaN cap layer samples with different *L*_GD_, there is a difference in μ_PCF_. This is because the additional scattering potential *V* (x, y, z) corresponding to APC is different for samples with different *L*_GD_. For the 2DEG under the gate, *V* (x, y, z) is expressed as follows [12]:

(17) $$\begin{array}{ll} V\left(x,y,z\right)= & -\frac{e}{4\pi \varepsilon_{s}\varepsilon_{0}}\int_{-L_{\mathrm{GS}}-\frac{L_{G}}{2}}^{-\frac{L_{G}}{2}}dx^{\prime }\int_{0}^{W}\frac{-\Delta \rho_{G}}{\sqrt{{\left(x-x^{\prime }\right)}^{2}+{\left(y-y^{\prime }\right)}^{2}+z^{2}}}dy^{\prime }\\ & -\frac{e}{4\pi \varepsilon_{s}\varepsilon_{0}}\int_{\frac{L_{G}}{2}}^{L_{\mathrm{GD}}+\frac{L_{G}}{2}}dx^{\prime }\int_{0}^{W}\frac{-\Delta \rho_{G}}{\sqrt{{\left(x-x^{\prime }\right)}^{2}+{\left(y-y^{\prime }\right)}^{2}+z^{2}}}dy^{\prime } \end{array}$$

where $\varepsilon_{s}\varepsilon_{0}$ is the dielectric constant. For G-C-Sample-2, because of its larger *L*_GD_ and the larger integration interval (*L*_G_/2, *L*_GD2_ + *L*_G_/2) of the second term in Formula (17), the *V* (x, y, z) obtained is stronger. Therefore, its scattering intensity for 2DEG is stronger, resulting in a smaller μ_PCF_. For A-C-Sample-1 and A-C-Sample-2, although there is a large difference in the corresponding integration intervals when calculating *V* (x, y, z), the APC of these two samples is small, and the PCF scattering effect is weak, so the integration interval has a minor impact. Therefore, the difference in μ_PCF_ between A-C-Sample-1 and A-C-Sample-2 is small. The AlN cap layer effectively mitigates the degradation in 2DEG mobility caused by variations in *L*_GD_ via suppressing PCF scattering.

## 4. Conclusions

In this work, we conducted a systematic investigation into how the AlN cap layer influences PCF scattering in AlGaN/GaN HFETs. By analyzing the electron mobility calculated based on electrical performance measurement data and scattering theory, we found that the AlN cap layer significantly suppresses the IPE in the barrier layer, primarily because of the stronger polarization and larger Young’s modulus of AlN relative to GaN, resulting in a reduced generation of APC under the gate. Less APC corresponds to a weaker PCF scattering potential. Meanwhile, the introduction of the AlN cap layer increases *n*_2DEG_, which enhances the Coulomb screening effect and helps reduce the intensity of PCF scattering. The effect of the AlN cap layer on *μ*_PCF_ is the result of the combined action of these two factors. Additionally, the mobility analysis of samples with different *L*_GD_ shows that devices with AlN cap layers exhibit better stability with respect to *L*_GD_ variations, indicating that the AlN cap layer helps mitigate the impact of variations in *L*_GD_ on 2DEG mobility. This study clarifies the mechanism of the influence of the AlN cap layer on PCF scattering and analyzes the effect of AlN cap layer on electron transport from the perspective of PCF scattering, providing a new theoretical basis for improving device performance by optimizing the material structure of the cap layer.

## Data Availability

The original contributions presented in this study are included in the article. Further inquiries can be directed to the corresponding author.
